# The role of cellular oxidative stress in regulating glycolysis energy metabolism in hepatoma cells

**DOI:** 10.1186/1476-4598-8-32

**Published:** 2009-06-05

**Authors:** Dong-yun Shi, Fei-zhou Xie, Chao Zhai, Jeremy S Stern, Yang Liu, Shan-lin Liu

**Affiliations:** 1 Department of Biochemistry and Molecular Biology, Shanghai Medical College of Fudan University, Free Radical Regulation and Application Research Center of Fudan University, Shanghai 200032, PR China; 2 Pharmaceutical Science Research Division, School of Health and Life Sciences, King's College London, London, SE1 9NH, UK; 3 Department of Neurology, St George's Hospital, London, SW17 0QT, UK; 4 Free Radical Life Science Research Center, Zhejiang University, Hangzhou 310027, PR China

## Abstract

**Background:**

The Warburg effect has been found in a wide spectrum of human cancers, however the underlying mechanisms are still unclear. This study aims to explore the role of cellular oxidative stress in relation to glycolysis and the Warburg effect in hepatoma cells.

**Methods:**

Various cell lines combining environmental hypoxia was used as an in vitro model to mimic tumor microenvironment in vivo. Superoxide dismutases (SOD) and xanthine oxidase (XO) gene transfection were used to produce various cellular redox levels. 2',7'-dichlorofluorescin (DCF) fluorescence and ESR spectrum were used to detect cellular reactive oxygen species (ROS).

**Results:**

We found that endogenous or exogenous interference with the cellular oxidative stress can sensitively regulate glycolysis and the Warburg effect in hepatoma cells. Hepatoma cells displayed a high level of free radicals compared to immortalized normal hepatocyte cells. Increasing the level of ROS stress in hepatoma cells can directly upregulate HIF-1 and activate glycolysis without requirement of a hypoxic condition. This explains the mechanism whereby aerobic glycolysis, i.e. the Warburg effect arises. Either endogenously upregulating SOD or exogenously administration with antioxidant can, through downregulating ROS level, effectively regulate energy pathways in hepatoma cells and can inhibit the growth of tumor cells and xenograft tumors.

**Conclusion:**

This study suggests that the Warburg effect was related to an inherently high level of cellular ROS and HIF-1. Hepatoma cells adaptation to hypoxia for survival and rapid growth exploits oxidative stress ectopically activated glycolysis to compensate the energy supply. This specific mechanism in which tumor cells through cellular oxidative stress activate glycolysis to meet their energy metabolism requirement could be exploited to selectively kill tumor cells.

## Background

In the presence of oxygen, normal cells completely oxidize glucose to CO_2 _and H_2_O, and generate ATP through aerobic oxidation. However, over 70 years ago, Warburg observed that cancer cells exhibit enhanced conversion of glucose to lactate (aerobic glycolysis) and depend heavily on the glycolytic pathway to meet their energy needs even in the presence of an adequate oxygen supply [1]. During the past decades, the Warburg effect has been found in a wide spectrum of human cancers, however the underlying mechanisms are still unclear.

Hypoxia in the tumor microenvironment is a common feature of solid tumors. Rapid growth of cancer cells and rapid expansion of the tumor mass usually leaves the generation of new vasculature lagging behind. The lack of oxygen delivery results in local ischemia and hypoxia of the tumor [2]. Such a hypoxic environment inside the tumor limits the availability of oxygen for use in mitochondrial respiration and production of ATP, and forces tumor cells to up-regulate the glycolytic pathway, in which oxygen is not required, as a main source of energy to maintain a sufficient energy supply for tumor growth [3,4].

Hypoxia is known to stimulate mitochondria to release ROS (mROS). Under hypoxic conditions, mitochondria participate in a ROS burst generated at complex III of the electron transport chain [5]. ROS is an important secondary messenger in signaling transduction [6,7]. The increased ROS in response to hypoxia can promote cancer cell survival and tumor growth through activating hypoxia inducible factor 1α (HIF-1α) [8].

Under normal conditions, intracellular ROS are maintained at a low level by various enzyme systems which maintain the in vivo redox homeostasis. Tumor cells usually have an unbalanced redox status, oxidative levels in tumor cells being relatively higher [9-11]. Moreover, moderately enhanced oxidative stress by slight upregulation of the ROS level can promote cancer cell growth but has no effect on normal cells [7,12]. This suggests that cancer cells are able to adapt to oxidative stress and that the survival microenvironment required for cancer cells and normal cells is different.

Given that tumor cells display the Warburg effect and that their survival relies on glycolysis to supply energy, it is of interest to study the role of oxidative stress in relation to the Warburg effect. Our previous study has already shown that hepatoma cells growth was dependent on cellular ROS level [13]. We have found that ROS could, through HIF-1, regulate gene expression involved in glycolysis in response to hypoxia [14]. Another recent study has also shown that the antitumorigenic effect of antioxidants is HIF-dependent [15]. These results suggest that ROS could through HIF-1 regulate glycolysis. However, why tumor cells rely on glycolysis in the presence of oxygen, i.e. the Warburg effect, and whether the cellular ROS are involved in regulation of the Warburg effect remains to be defined. Prior studies have shown that the cellular redox microenvironment can be altered through transfection by MnSOD genes [16]. In the present study, we tested the effects of different cellular status on the Warburg effect. High oxidative level was achieved by either exposure to hypoxia or overexpression of xanthine oxidase, and a decreased oxidative level was achieved by antioxidant treatment or overexpression of MnSOD. For the first time, we found that manipulating cellular oxidative stress microenvironment in hepatoma cells can regulate the Warburg effect.

## Results

### Cancer cells under hypoxic stress exhibit a survival advantage paralleled by compensatory upregulated glycolysis

**Figure 1:**
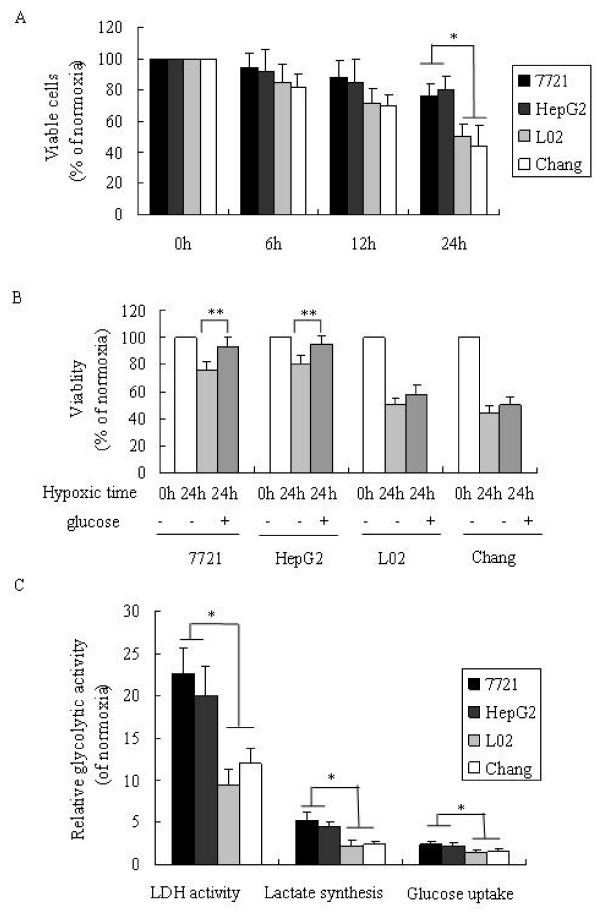
**Comparison of hepatoma and hepatocyte cells in response to hypoxia**. (a) Survival ratio of hepatoma and hepatocytes cells in response to hypoxia. Hepatoma cell lines SMMC7721 and HepG2, and hepatocyte cell lines L02 and Chang were cultured under 2% O_2 _hypoxic condition for indicated times, and viable cells were assessed by live cell counting using trypan blue exclusion method. Data were expressed as relative to control values at normaxia incubated for the same period. (b) Glucose increases cell survival in response to hypoxia. Cells were pretreated with 5 mmol glucose and cultured under 2% O2 hypoxic condition for 24 h. (c) Glycolytic activity changes in response to hypoxia. Cells were cultured for 24 h under 2% O2. LDH activity, cellular lactate synthesis and glucose uptake were measured as described in materials and methods. LDH activity levels, cellular lactate production and glucose consumption are expressed relative to control values at normoxia. Control value is presented as 1. *, P < 0.05, difference between hepatoma cells and hepatocyte cells (a, c); **, P < 0.05, versus cells in the absence of glucose treatment (b). Results are means ± S.D. of four independent experiments.

We first examined the effects of hypoxia on hepatoma cells and immortalized normal liver cells. L02 and Chang liver cells are immortalized non-tumor cell line derived from normal liver tissue and are considered an in vitro model of nonmalignant liver [17-20]. We compared the differential susceptibility of human hepatoma cell lines SMMC-7721 and HepG2, and human normal liver cell lines L02 and Chang subject to 2% O_2 _severe hypoxic condition. The survival of human hepatoma cells and hepatocyte cells in response to hypoxia were both inhibited in a time-dependent fashion that decreased with the hypoxia time (Fig. 1a). The survival rate of hepatoma cells was significant higher than hepatocyte cells during 24 h hypoxia. This finding suggests that hepatoma cells have a higher tolerance to hypoxia than hepatocyte cells. The decreased survival ratio induced by hypoxia in hepatoma cells and hepatocyte cells was attenuated by pre-treatment with 5 mM glucose in both cases (Fig. 1b). Both SMMC-7721 and HepG2 cells appear more sensitive to glucose treatment than hepatocyte cells. This suggests hepatoma cells rely more heavily on glycolysis. In order to verify that hepatoma cells 7721 and HepG2 have a higher glycolytic ability, we further examined the activity of lactate dehydrogenase (LDH), a key enzyme involved in glycolysis, and lactate synthesis and glucose uptake in cells. As illustrated in Fig. 1c, cells under hypoxic condition produced a significantly greater level of LDH activity, lactate production and glucose consumption than under normoxia. Hepatoma cell lines produced about 20-fold LDH activity and hepatocyte cell lines produced about 10-fold LDH activity. LDH activity appears to be the most sensitive parameter reflecting glycolytic activity. The increased glycolytic activity induced by hypoxia was also shown to be clearly greater in hepatoma cells than that in hepatocyte cells. This also suggests that hepatoma cells benefit from up-regulating glycolysis in response to hypoxia compared to hepatocyte cells.

### The involvement of ROS in regulating glycolysis under hypoxia

**Figure 2:**
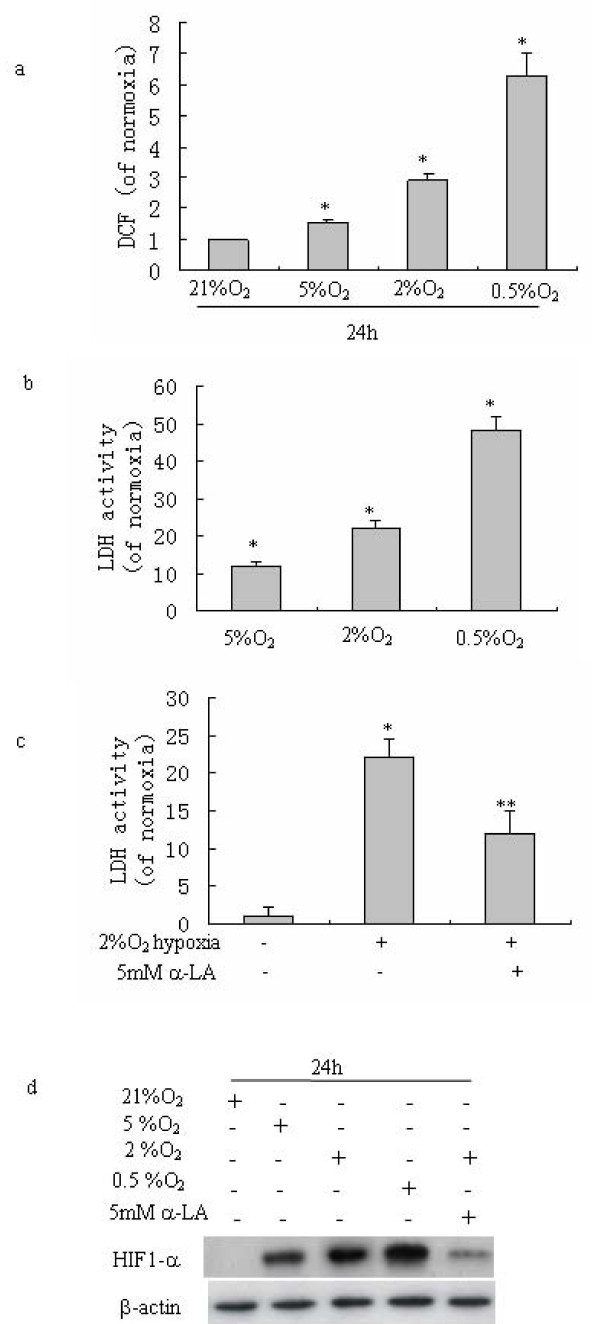
**ROS up-regulate glycolysis in hepatoma cells under hypoxia**. (a) Hypoxia stimulates ROS generation. SMMC-7721 hepatoma cells were exposed to 0.5% O_2_, 2% O_2_, and 5%O_2 _for 24 h, and ROS generation was determined by DCF fluorescence using flow cytometry. (b) LDH activity increases in response to hypoxia. SMMC-7721 hepatoma cells were incubated for 24 h under indicated hypoxic conditions, and LDH activity was measured as described in "Materials and methods". (c) The effect of antioxidant α-LA on LDH activity under hypoxia. Cells were pre-treated with or without 5 mmol/L α-LA and exposure to 2% O2 for 24 h. Scavenging ROS by α-LA attenuates hypoxia-induced LDH activity. (d) ROS is involved in hypoxia-induced HIF-1α stabilization. Cells were subjected to different hypoxic condition for 24 h, in some cases cells were pre-treated with 5 mM α-LA. Nuclear HIF-1α levels were assessed by Western blot as described in Materials and methods. β-actin shows the internal reference for semiquantitative loading in each lane. The present blots are representative of three experiments. Columns, data were normalized to 21% O_2 _normoxia controls (a, b, c) and represent the means ± S.D. of four independent experiments. *, P < 0.05 versus normoxia. **, P < 0.05 versus cells in the absence of α-LA treatment under 2% O_2 _hypoxia.

It is known that hypoxia can stimulate ROS generation. We examined the ROS level in SMMC-7721 cells subjected to 0.5%, 2%, 5% O_2 _of hypoxia. The levels of ROS monitored using DCF fluorescence (DCFH oxidation) increased with the degree of hypoxia (Fig. 2a). Meanwhile cells showed no change in fluorescence when labeled with the oxidation insensitive probe. The increased ROS was paralleled by upregulated LDH activity (Fig. 2b). α-lipoic acid (α-LA) is a powerful antioxidant, it can scavenge various ROS [21]. Pre-treatment of cells with 5 mM α-LA prior to hypoxia resulted in a decrease in LDH activity (Fig. 2c). This suggests that in hepatoma cells ROS is involved in up-regulating glycolysis under hypoxic conditions.

Studies to identify the intracellular oxygen sensor suggest that HIF1-α is a central regulator of the response to hypoxia [22]. Hypoxia induced ROS has been reported to be able to stabilize HIF1-α [5]. Western blot analyses of nuclear extracts revealed that levels of HIF1-α protein were enhanced by the degree of hypoxia (Fig. 2d). The HIF1-α activation was paralleled by ROS generation (Fig. 2a). Hypoxia-induced HIF1-α was suppressed by preincubation with 5 mM α-LA (Fig. 2d). These findings suggest the involvement of ROS generation in HIF1-α stabilization by hypoxia, and indicate that hypoxia induced ROS could regulate glycolytic gene expression by activating HIF1-α.

### Enhanced endogenous ROS caused an increase in glycolytic activity

**Figure 3:**
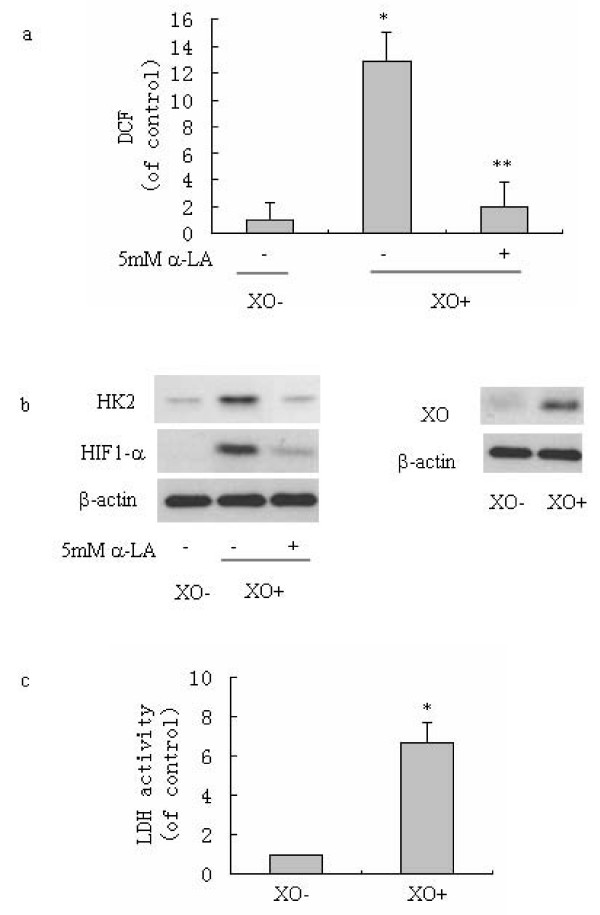
**Enhanced endogenous ROS up-regulate glycolytic activity**. (a) Quantification of ROS levels in SMMC-7721 cells in comparison with their XO-transfected cells. SMMC-7721 cells were transfected with a void vector (XO-) or a vector expressing XO (XO+) as described in "Materials and methods". In some cases XO+ cells were treated with 5 mM α-LA for 24 h. ROS levels were measured by DCF fluorescence using flow cytometry as described in "Materials and methods". Data were expressed relative to control SMMC-7721 cells (XO-). (b) Enhanced XO expression caused an elevated protein level of HIF1-α and HK2. In some cases XO+7721 cells were treated with 5 mM α-LA for 24 h. XO, HIF1-α and HK2 protein levels were assessed by Western blot as described in "Materials and methods". The present blots are representative of three experiments. (c) Comparison of glycolytic enzymes activity (LDH activity) in SMMC-7721(XO-) and XO-transfected 7721 cells (XO+). LDH activity levels are expressed relative to control SMMC-7721 cells (XO-). * P < 0.05 versus 7721 cells (XO-) in the absence of α-LA treatment (a, c); **, P < 0.05 versus XO+7721 cells in the absence of α-LA treatment (a). Columns: means ± S.D. of four independent experiments.

If hypoxia induced HIF1-α and glycolytic enzyme gene expression is ROS-dependent, ROS should activate gene expression by activating HIF1-α expression in normoxia. To test this hypothesis, an XO-transfected SMMC-7721 hepatoma cell line which has elevated ROS was used to assess the effects of endogenous ROS on HIF1-α expression. As illustrated in Fig 3a, the pLNCX2-XO-7721 cell line has elevated ROS levels, as indicated by DCF fluorescence. The Western blot showed that XO-transfected 7721 cells (XO+) have increased XO and HIF1-α expression (Fig 3b). Antioxidant α-LA treatment reduced ROS (Fig. 3a), and also attenuated HIF1-α expression (Fig. 3b). This result confirms that HIF1-α can be activated in a ROS-dependent manner. We next tested if ROS regulates expression of hypoxia-induced genes required in glycolysis. Hexokinase (HK) is a key enzyme involved in glycolysis. We analyzed HK2 protein expression and LDH activity in XO-transfected 7721 cells. The results show that XO-transfected 7721 cells have increased HK2 protein level and LDH activity compared to control 7721 cells (Fig. 3b and 3c). This suggests that ROS can also stimulate glycolytic activity independent of hypoxia and that aerobic glycolysis in tumor cells is related to the inherently high level of ROS in tumor cells.

### Reduction of cellular ROS level inhibits glycolytic activity

**Figure 4:**
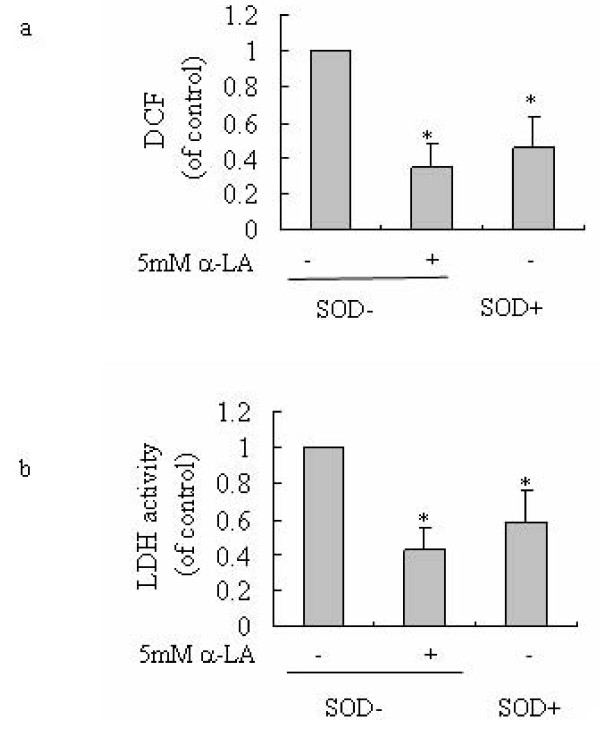
**Reduction of cellular ROS decreases glycolytic activity**. (a) Cellular ROS was decreased either by ROS scavenging or by MnSOD transfection. (b) LDH activity was inhibited by reduction of cellular ROS. SMMC-7721 cells transfected with a void vector (SOD-) or with a vector expressing SOD (SOD+) were incubated for 24 h in the presence or absence of 5 mM α-LA. ROS levels (a) were determined by DCF fluorescence, and LDH activity levels (b) were measured as described in "Materials and methods". Results were expressed relative to control SMMC-7721 cells (SOD-) in the absence of α-LA treatment. * P < 0.05 versus control SMMC-7721 cells (SOD-) in the absence of α-LA treatment. The values represent the means ± S.D. from three separated experiments.

The above observations show that increased ROS benefits cancer cells through up-regulation of glycolysis. We further tested whether the ROS induced oxidative cellular status is critical for the Warburg effect. To test this possibility, we used the antioxidant α-LA to reduce the cellular oxidative status and examined the glycolytic activity of cells. As shown in Fig. 4a and 4b, incubation of SMMC-7721 cells with 5 mM α-LA caused a decrease of ROS and down-regulation of LDH activity, suggesting the reliance of glycolytic activity on ROS.

In order to confirm the dependence of glycolysis activation on cellular oxidative stress status, we further established a sense MnSOD transfected cell line, which has an endogenous low level of ROS (Fig. 4a). Consistent with the exogenous antioxidant treatment, the sense MnSOD transfected cells have a low level of LDH activity (Fig. 4b). These findings suggest that the specific higher level of oxidative states within cancer cells is important for the Warburg effect.

### Comparison of cellular ROS level and relative glycolytic activity in hepatoma cells and immortalized normal liver cells

**Figure 5:**
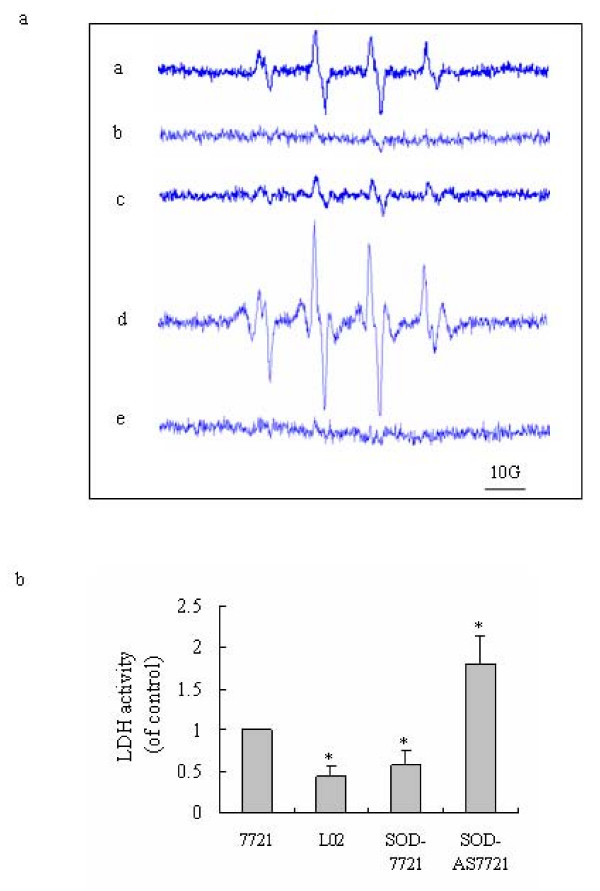
**Cellular ROS levels and relative LDH activities**. (a) ROS production monitored by ESR spectroscopy. 3 × 10^6 ^cells/ml SMMC 7721 hepatoma cells (Panel a) or L02 liver cells (Panel b), or SOD-transfected 7721 cells (Panel c), or antisense-SOD-transfected 7721 cells (Panel d) were incubated with BMPO (100 mM). ROS production was assessed by ESR spectroscopy as described under "Materials and methods". In panel (e) SMMC-7721 cells were treated as in panel (a) with SOD (100 U/mL). The present spectrums are representative of three experiments. (b) Comparison of cellular LDH activity. LDH activity levels in SMMC-7721 cells, L02 cells, SOD-transfected 7721 cells (SOD-7721) and antisense-SOD-transfected 7721 cells (SOD-AS7721) were measured as described in "Materials and methods". LDH activity was expressed relative to control SMMC-7721 cells. Columns: * P < 0.05 versus control SMMC-7721 cells. The values represent the means ± S.D. from four independent experiments.

These findings suggest that a higher cellular oxidative stress level favored activation of glycolysis in cancer cells. ROS usually have a very short half-life, and can be rapidly degraded. In order to validate ROS detection using DCF and understand the difference in ROS levels and types in various cell lines, cellular ROS were further examined by the ESR method using BMPO spin trap. As shown in Fig. 5a, 7721 hepatoma cells produced a robust BMPO-OH signal (Fig. 5a.a), however this BMPO-OH signal was undetectable in the immortalized normal L02 liver cells (Fig. 5a.b). In SOD-7721 cells this free radical signal was attenuated (Fig. 5a.c), and in SOD-AS7721 cells this free radical signal was enhanced (Fig. 5a.d). The BMPO-OH adduct signal was completely quenched by pre-administration of cells with 100 U/mL of SOD, a specific superoxide anion scavenger (Fig. 5a.e). This suggests that the main source of free radicals captured in SMMC-7721 hepatoma cells was superoxide anion free radical. The ESR experiment further confirmed that hepatoma cells were under a relatively higher level of ROS stress.

Meanwhile, the measurement of relative LDL activity showed that cellular glycolytic activity is related to the ROS stress level. Paralleled with the change of ROS level (Fig. 5a), LDH activities in each cell lines displayed in different levels (Fig. 5b). SOD-AS7721 which has the highest ROS level, accordingly displayed the highest LDH activity. However, SOD-7721 and L02 cells which have relative lower levels of ROS, exhibited significantly lower levels of LDH activity compared to SMMC-7721 cells. This confirms that ROS can regulate glycolytic activity independently of hypoxia.

### Decreasing cellular oxidative stress inhibits tumor growth in vitro and in vivo

**Figure 6:**
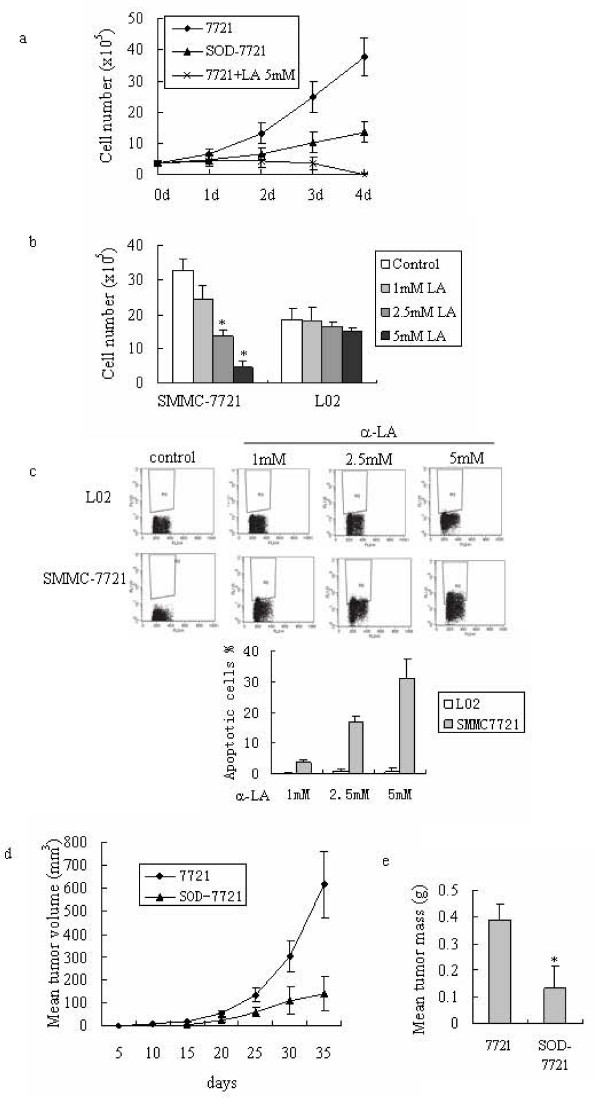
**Reduction of ROS inhibits the growth of tumor cells and xenograft tumors**. (a) Reduction of ROS inhibits SMMC-7721 hepatoma cells growth. SMMC-7721 hepatoma cells were administrated with 5 mM α-LA or transfected with MnSOD gene (SOD-7721 cells) to downregulate cellular ROS. Cells were harvested at day 1, 2, 3 and 4. Cell growth was monitored by live cell counting using trypan blue exclusion method. (b) Comparison of different doses of α-LA on the growth of SMMC-7721 hepatoma cells and L02 immortalized normal liver cells. Cells were treated with 1, 2.5, and 5 mM α-LA, and harvested at 3 days after treatment. Cell growth was monitored by live cell counting using trypan blue exclusion method. (c) α-LA induces apoptosis in SMMC-7721 human hepatoma cells. Cells were cultured for 3 days with 1 mM, 2.5 mM and 5 mM α-LA. Apoptosis was evaluated by TUNEL as described in methods. (d) Tumor sizes in nude mice bearing human tumor xenografts. Nude mice were injected with SMMC-7721 cells transfected with a void vector or a vector expressing SOD (SOD-7721) as described in the methods. Tumor sizes were measured in three directions every 5 days. (e) Tumor masses in nude mice bearing human tumor xenografts. After 5 weeks, mice were killed and tumor masses were weighted. For cell experiments, the values represent the means ± S.D. from three separated experiments. * p < 0.05 compared with SMMC-7721 cells. For animal experiments, results were expressed as means ± S.D., n = 10 per group. * p < 0.05, compared with mice bearing SMMC-7721 cells.

We further tested whether the change of cellular oxidative states could influence tumor growth. Either antioxidant α-LA treatment or MnSOD transfection were used to scavenge cellular ROS and decrease the cellular oxidative stress level. As shown in Fig. 6a, antioxidant interference and SOD transfection both significantly inhibit hepatoma cells growth. Combining the results from Fig. 4, these results suggest that decreasing cellular oxidative stress could through inhibiting glycolytic activity in tumor cells inhibit tumor cells growth. Moreover, α-LA was shown to inhibit SMMC7721 hepatoma cells growth in a dose-dependent manner (Fig. 6b), and in a certain dose (2.5 mM and 5 mM) α-LA induced hepatoma cell apoptosis (Fig. 6c). However, the same concentration of α-LA has no obvious effect on immortalized normal L02 liver cells. This suggests that oxidative stress in hepatoma cells can be exploited to selectively kill cancer cells.

In keeping with the results of the in vitro experiments, nude mice injected with SMMC-7721 hepatoma cells formed bigger xenograft tumors than those injected with SOD-7721 cells. As shown in Fig. 6d, mice injected with SMMC-7721 cells start to form visible tumor by 5 days (about 1 mm) with subsequent rapid growth. Mice injected with SOD-7721 cells start to form tumor at about 10–15 days, and the tumors grow slowly. Apart from SOD-cells resulting in a delay in tumor formation in nude mice, it also gives rise to significantly smaller tumors than SMMC-7721 cells as judged by total tumor mass (Fig. 6e). These results from xenograft tumors confirm that reduction of oxidative level in tumor cells could inhibit tumor growth.

## Discussion

The Warburg effect is the basis for the widespread application of positron emission tomography, established in the mid 1990s, in which a glucose analog tracer is used to differentiate normal and tumor tissue, as tumor tissue takes up glucose more avidly [23]. The Warburg effect has been observed in various tumor cells, including solid tumors and leukemia, and is recognized to represent a prominent metabolic characteristic of malignant cells [24]. At present several possible mechanisms have been proposed to explain this metabolic difference of tumor cells. These mechanisms include mitochondrial malfunction [25,26], oncogenic transformation [27,28], and tumor microenvironment [29]. However, the exact mechanisms causing tumor cells to use this primitive and less energy-efficient pathway to generate ATP to maintain rapid growth are still to be explained.

It is known that hypoxia is one of the distinguishing and near-universal hallmarks of cancer growth. The cellular environmental hypoxic conditions could force cancer cells to use glycolysis to generate ATP to meet their energy requirement. Environmental hypoxia has been used as an in vitro model to study the Warburg effect in vivo [30,31]. We exposed cultured hepatoma cells to environmental hypoxia and analyzed changes in glycolytic activity in response to hypoxia. We compared the effect of hypoxia on hepatoma cells and immortalized normal liver cells. L02 and Chang liver cell lines were used as the nontransformed normal cells to compare their biological features with the control malignant cells [18-20]. Our results show that hepatoma cells have a higher tolerance to hypoxia than liver cells and that hepatoma cells under hypoxic stress exhibit a survival advantage compared to liver cells (Fig. 1). Pretreatment with glucose before hypoxia significantly increased the survival of heaptoma cells. These results indicate that cancer cells rely more heavily on glycolysis than normal cells. The ability of hepatoma cells to upregulate glycolysis in response to hypoxia was further confirmed by the finding of increased lactate synthesis and glucose consumption, and enhanced activity of LDH, a key enzyme in glycolysis. It is known that normal cells mainly rely on aerobic oxidation to meet the energy requirement for growth. Under hypoxia conditions glycolysis can be correspondingly upregulated in normal cells via the Pasteur effect. However, in response to prolonged hypoxia (24 h-hypoxia) (Fig. 1a), as the excess hypoxia has impeded aerobic oxidation, upregulated glycolysis is insufficient to compensate the energy deficit, which will lead to damage of normal cells and induce apoptosis. In the case of cancer cells under the same hypoxic conditions their upregulated glycolytic activity can be further activated extending a survival advantage to tumor cells in response to hypoxia compared to normal cell lines.

We further addressed the relationship between upregulated gylcolysis and ROS in response to hypoxia in hepatoma cells. It is known that hypoxia can stimulate ROS generation. Although the stimulation of ROS during hypoxia has been already described, how ROS relate to glycolysis in cancer cells under hypoxia is not clear. Our studies show that the dependency on glycolysis in hepatoma cells was actually related to ROS (Fig. 2). ROS were increased with the degree of hypoxia (Fig 2a). The increased ROS were associated with upregulated glycolytic activity (Fig 2b). A decline of ROS caused by antioxidant pre-treatment before hypoxia in hepatoma cells resulted in a decrease of LDH activity (Fig. 2c). These results suggest that ROS are involved in upregulating glycolytic activity in hypoxic hepatoma cells.

In order to understand the mechanism whereby ROS upregulates glycolysis under hypoxia, we investigated the role of ROS in regulation of HIF1-α. Hypoxia is known to be capable of activating HIF1-α. HIF1-α is a key transcription factor which can up-regulate a series of genes involved in glucose transport, glycolysis, erythropoiesis, angiogenesis, vasodilation, and respiratory rate [32,33]. We observed that hypoxia increased the both of ROS level and HIF1-α activity. When ROS level was reduced by antioxidant α-LA pre-treatment, hypoxia induced HIF1-α expression was downregulated (Fig. 2d). This result suggests that ROS is important for the up-regulation of HIF-1.

If hypoxia induced HIF1-α expression is mainly dependent on ROS, ROS should also be able to directly activate HIF1-α expression without hypoxic condition. Indeed, we showed that when ROS were increased by XO gene transfection, which altered the cellular microenvironment in SMMC-7721 cells, HIF1-α protein expression was up-regulated. The HIF1-α targeted glycolytic enzyme such as HK2 and LDH were accordingly increased in XO-7721 cells. Antioxidant treatment could abolish the increase (Fig. 3). It is known that HIF1-α is continuously synthesized and degraded under non-hypoxic conditions due to rapid hydroxylation by proly 4-hydroxylase (PHD). Therefore normally the level of HIF1-α is very low. Recent studies show that various oxygen species can promote HIF-1α stabilization by inhibiting PHD [34,35]. HIF-1 was shown to be able to regulate glycolysis and respiration through mediating expression of pyruvate dehydrogenase kinase [36]. Therefore, the excessive ROS may, through inhibiting PHD, promote HIF-1α stabilization, causing the high level of HIF1-α, which results in the up-regulation of glycolysis.

Increasing endogenous ROS by gene transfection can cause increased glycolytic activity (Fig. 3). This suggests a critical role of the cellular oxidative stress in the Warburg effect. Decreasing cellular ROS level either by antioxidant, or by MnSOD transfection can decrease glycolytic activity (Fig. 4). This further suggests the reliance of the Warburg effect on ROS. This characteristic is consistent with our previous finding that tumor cells growth is dependent on ROS [7,13]. Therefore a cellular oxidative stress microenvironment is important for the tumor specific Warburg effect in hepatoma cells, glycolysis could be activated in a ROS dependent but hypoxia-independent manner. This also could explain the mechanism whereby tumor cells rely on aerobic glycolysis even in the present of oxygen so displaying the Warburg effect.

Although growing evidences suggest that cancer cells are under increased ROS and ROS-induced oxidative stress compared to normal cells, conflicting results regarding redox states in tumor cells have also been reported [37-39]. Our results suggest that SMMC-7721 hepatoma cells have a much higher level of oxidative stress than L02 normal liver cells. These results were validated by directly detecting free radical signal in cellular microenvironment using ESR spectrum method. In the present study, using BMPO spin trap and SOD interference, free radical signals in various hepatoma cell lines were captured and the main source of free radicals was confirmed to be superoxide anion free radical (Fig. 5a). However, no obvious free radical signal can be seen in normal L02 liver cells. HepG2 hepatoma cells were found to have similar higher level of oxidative stress compared to Chang liver cells (data were not shown here). We have also measured other type of tumor cells such as gastric cancer cells, they presented the same characteristic. Furthermore, the cellular oxidative stress increased according to a decreasing degree of differentiation in four gastric cancer cell line [40]. This suggests that a higher oxidative stress level in malignant tumor cells is not just a specific feature of a single cell line. The conflicting results seen in other studies might be due to experimental models or failure to assess cellular ROS level.

Our results also suggest that the immortalized normal liver cell lines behave similarly to normal cells rather than cancerous cells. As shown in Fig. 5a, the L02 cells have relatively lower intrinsic ROS levels, like normal liver cells. Meanwhile they also have lower glycolytic activity (Fig. 1c, Fig. 5c), a property which is also shared by normal liver cells. Our results also suggest that the Warburg effect is related to a high level of cellular ROS and HIF-1 in tumor cells, which is different to the phenomena seen in the normal cell lines. Our results also show that antioxidant α-LA can through inhibiting glycolytic activity inhibits hepatoma cell growth, and at a certain dose it can further induce apoptosis of hepatoma cells. However, the same treatment was not harmful to L02 liver cells. As ROS can through HIF-1 increase glycolysis, a certain ROS level will benefit cancer cell growth. Scavenging ROS can induce apoptosis through inhibiting cancer cell growth. However, the L02 normal cell line under normoxia conditions has lower a ROS level and lower glycolytic activity. Energy generation is mainly through oxidative phosphorylation, and so these cells are relatively insensitive to ROS scavengers.

In addition to its antioxidant properties α-LA is also known to be a co-factor of many enzymes such as pyruvate dehydrogenase complex involved in the oxidative phosphorylation. The effect of α-LA could in part be due to its non-antioxidant function. Taking into consideration that many tumor cells have dysfunctional mitochondria resulting in dysfunctional oxidative phosphorylation and upregulated glycolysis, α-LA may not benefit cancer cell through increasing oxidative phosphorylation. Contrarily, LA may through scavenging ROS inhibit glycolysis and inhibit the energy supply of cancer cells. We have tested other antioxidants treatment such as NAC and Resveratrol and found similar (data were not shown here). This suggests that the antioxidant function of α-LA may play a primary role.

The xenograft tumor studies also have shown similar results. Nude mice injected with SOD-7721 cells showed a delay in tumor formation as compared with those injected with SMMC-7721 cells. In recent years, human tumor xenografts have been successfully inhibited through decreasing HIF-1 level by using exogenous antioxidants [15], and several antioxidant trials have been conducted against cancer [41]. In these trials antioxidants were applied as a supplemental treatment. However, the overall effects in trials are not significant, in some cases negative effects have also been reported [42]. These results may be explained by different redox status in different cells. As we demonstrate in this paper, effective inhibition of tumor cell growth may be achieved by altering the cellular oxidative stress. It is known that malignant cells of different cancer types exhibit heterogeneity in levels of oxidative stress, associated with various expression levels of SOD and other antioxidant enzymes [37-39,43]. Patients usually also have various degrees of oxidative stress in vivo according to their cancer stage [44]. Therefore, it may be critical to effectively monitor cellular ROS level and the associated energy supply pathway in the microenvironment of tumor tissue and tumor cells during administration of antioxidants to treat cancer.

## Conclusion

Taken together, these results suggest that altering cellular redox level in hepatoma cells can modulate the tumor specific Warburg effect, and that the cellular oxidative stress microenvironment is important for hepatoma cells to rapid growth, which may be applicable for cancer in general. The mechanism allowing hepatoma cells through oxidative stress to ectopically activate glycolysis could be exploited to selectively kill tumor cells through interference with energy pathways.

## Methods

### Cell culture and hypoxia exposures

The human hepatoma cell line SMMC-7721 was obtained from Shanghai Institute of Cell Biology. The human hepatoma cell line HepG2, and human hepatocyte cell lines L02 and Chang were generously provided by Institute of Zhongshan Hospital. Cells were maintained in RPMI-1640 (Gibcol BRL) supplemented with 10% heat-inactivated (56°C, 30 min) fetal bovine serum, L-glutamine (2 mM), penicillin (100 units/mL), streptomycin (100 μg/mL), at 37°C in a humidified environment of 5% CO_2_-95% air. After cells were serum starved for 24 hours, cell culture media was replaced by hypoxic medium (obtained by bubbling the serum free RPMI medium or serum free RPMI medium supplement with glucose for 4 hours with 0.5% O_2_:94.5% N_2_:5% CO_2_, or 2% O_2_:93% N_2_:5% CO_2_, or 5%O_2_:90% N_2_:5% CO_2 _gas mixture). The flasks were then gassed with appropriate hypoxic gas mixture and incubated for indicated time at 37°C in a closed system.

### Plasmids and transfection

For overexpression of MnSOD to downregulate ROS levels, or inhibtion of MnSOD to increase ROS level, plasmids containing sense or antisense cDNA of human MnSOD were used. pHβ A-SOD(+) or pHβ A-SOD(-) plasmids (kindly provided by Professor Kunitaka Hirose) were transfected into SMMC-7721 cells and establish human SMMC-7721 hepatoma cell lines with stable expression of MnSOD (represent as SOD-7721 cells) or with suppressed expression of MnSOD (represent as SOD-AS7721) using a standard method as described before [7]. For overexpression of XOR to enhance ROS levels, a retroviral construct encoding human xanthine oxidase (XO) cDNA (*hXO*) was used to produce SMMC-7721 cells overexpressing hXO [45]. A pLNCX2-XO construct (kindly provided by Professor Pin XU) was infected into SMMC-7721 cells, and established a human SMMC-7721 hepatoma cell line with stable expression of XO (represent XO-7721 cells) [45].

### Assessment of cell growth, cell survival rate and apoptosis

Cells were trypsinized with 0.25% trypsin and harvested at different time point. Cell number was counted using a haemocytometer by adding 0.2% trypan blue which stains the cytoplasm of dead cells but not live cells. Live cells were counted using this trypan blue exclusion method. Cell survival rate were calculated according to following formula: Cell survival rate(%) = number of survival cells/number of total cells (*100%). SMMC-7721 cell apoptosis was assessed by terminal deoxynucleotidyl transferase (Tdt) mediated dUTP Nick End Labelling (TUNEL) analysis using flow cytometry as descibed before [46].

### Nuclear extracts and Western blotting analysis

Nuclear and cytosolic fractions were isolated using NE-PER Nuclear and Cytoplasmic Extraction Reagents according to the instructions of the manufacturer (Pierce, Rockford, IL). Whole-cell lysates were prepared and Western blotting was performed as previous described [46]. Anti-HIF-1α monoclonal Antibody (BD Transduction Labs), anti-XO antibody (NeoMArkers), anti-hexokinase 2 antibody and anti-horseradish peroxidise (HRP)-conjugated antibody (Santa Cruz Biotechnology) were used for immunoblot assays.

### Measurement of reactive oxygen species

Intracellular ROS was assessed using oxidation sensitive probe 2',7'-dichlorodihydrofluorescein diacetate (DCFH) (San Diego, CA) as described [46] and using electron spin resonance (ESR) spectroscopy. In the DCFH method, the oxidation insensitive 2, 7-dichlorofluorescein diacetate (DCF) was used as a control to ensure that changes in uptake, ester cleavage, and efflux of the probe had not occurred. DCF fluorescence was determined by using 0.5 × 10^6 ^cells with a FACSCalibur (excitation wavelength, 488 nm; emission wavelength, 515–545 nm; Becton Dickinson). For each sample 10000 events were collected.

Spin trapping agent combined with ESR spectroscopy can directly assess cellular ROS. 10 μl spin trap 5-tert-butoxycarbonyl 5-methyl-1-pyrroline N-oxide (BMPO, 100 mM, synthesized in our group) was added to a 90 μl cell suspension (3 × 10^6 ^cells/ml in PBS, pH 7.4), and transferred to a flat quartz ESR aqueous cell. ESR measurements were carried out at room temperature using a Bruker-IBM ER 200D-SRC spectrometer equipped with an X-band (Germany). Studies were performed using the experimental conditions as previously described [13]. SOD (100 U/ml) was added to cell suspension as indicated and analyzed as described above.

### Measurement of glycolytic activity

The cellular glycolytic activity was determined by measuring the activity of key enzyme lactate dehydrogenase, and glucose uptake and lactate production. Lactate dehydrogenase catalyzes the reaction: L-lactate + NAD+ in equilibrium pyruvate + NADH. The activity of lactate dehydrogenase (LDH) was monitored spectrophotometrically by measuring the increase in NADH at 340 nm produced in the lactate-to-pyruvate reaction [47]. Lactate concentration in the culture medium was measured using commercial chromatometric kits from Sigma. For cellular glucose uptake, cells were incubated with glucose-free RPMI-1640 with 1 μCi 2-deoxy-[^3^H]-D-glucose for 60 minutes. Then cells were washed three times with ice-cold PBS. The radioactivity in the cells pallets was quantified by liquid scintillation counting.

### Tumor formation in nude mice

Subconfluent cells were detached and resuspended in the media at a density at 2 × 10^7^/ml. Male nude mice between four to six weeks old (BALB/c, nu/nu, from Experimental Animal Center of Shanghai, China Academy of Sciences), weighting 18–20 g, were injected subcutaneously with 0.2 ml of SMMC7721 cells or SOD-7721 cells respectively at two different sites. The tumor formation was assessed every 5 days. The sizes of the subcutaneous tumor were measured in three directions every 5 days. Five weeks after injection, mice were killed by cervical dislocation, the tumors were dissected and weighed.

### Statistical Analysis

Data are given as mean ± S.D. of three to five individual experiments. Comparisons between means were done by using Student's *t *test for paired data using Microsoft EXCEL software.

## Abbreviations

α-LA: α-lipoic acid; BMPO: 5-tert-butoxycarbonyl 5-methyl-1-pyrroline N-oxide; DCF: 2,7-dichlorofluorescein diacetate; DCFH: 2',7'-dichlorodihydrofluorescein diacetate; ESR: electron spin resonance; HK: hexokinase; LDH: lactate dehydrogenase; ROS: recative oxygen species; SOD: superoxide dismutases; XO: xanthine oxidase.

## Competing interests

The authors declare that they have no competing interests.

## Authors' contributions

DS designed and performed the research, analyzed the data, and wrote the paper; FX and CZ performed the part research; JS analyzed the data, and helped in drafting the manuscript. YL helped in the ESR detection. SL designed the research, analyzed the data, and revised the manuscript. All authors approved the final version of the manuscript.
